# Electrophysiological Measurement of Emotion and Somatic State Affecting Ambiguity Decision: Evidences From SCRs, ERPs, and HR

**DOI:** 10.3389/fpsyg.2020.00899

**Published:** 2020-05-14

**Authors:** Fuming Xu, Long Huang

**Affiliations:** ^1^School of Education Science, Nanning Normal University, Nanning, China; ^2^School of Psychology, Jiangxi Normal University, Nanchang, China; ^3^School of Humanities and Management, Wannan Medical College, Wuhu, China

**Keywords:** somatic marker hypothesis, Iowa gambling task, skin conductance responses, event-related potentials, heart rate

## Abstract

Twenty-three years ago, the Somatic Marker Hypothesis (SMH) proposed by Damasio was introduced to explain the role of emotion in decision-making, and provided a unique neuroanatomical framework for decision-making and its influence by emotion. The core idea of the SMH is that decision-making is a process that is affected by somatic state signals, including those that express themselves in emotion and feeling. In order to verify the SMH, the Iowa Gambling Task (IGT) was originally designed by Bechara et al. and the skin conductance responses (SCRs) was recorded during the IGT. The initial confirmatory results showed that normal subjects would generate anticipatory SCRs when they received reward or punishment, but patients of the VMPFC lesion entirely failed to generate anticipatory SCRs prior to their selection of a card. With the further development of the SMH–related researches, other electrophysiological methods of measuring somatic state was gradually used to test the SMH, including event-related potentials (ERPs), and heart rate (HR). In this mini review article, we summarize the extant electrophysiological research on the SMH and decision-making under ambiguity, propose an integrative perspective for employing different electrophysiological measurement methods, and indicate the application of electrophysiological measurement based on the SMH in daily social decision-making.

## Introduction

Emotion is considered a destructive factor in the cognitive process for a long time (Reimann and Bechara, 2010). However, twenty-three years ago, neuroscientist Antonio Damasio proposed the Somatic Marker Hypothesis (SMH) and introduced it to explore decision-making under ambiguity (Bechara et al., 1994; Damasio, 1994). The positive role of emotions in the decision-making process is gradually being valued by researchers. The consensus reached in previous studies is that emotions unrelated to current decision-making tasks can interfere with the decision-making process. But emotions related to current tasks, especially in complex decision-making under ambiguity, can guide decision makers avoid disadvantageous choices or situations and instead consider advantageous choices or situations (Bechara and Damasio, 2005). The SMH suggested that positive or negative emotional responses from external or internal stimuli can activate changes in the peripheral and central nervous systems of somatic, producing positive or negative somatic markers (e.g., heart rate, blood pressure, heart rate) that characterize emotional and emotional responses, and then guiding decision making. Whether you realize it or not, the somatic markers will always affect the decision-making at the unconscious level (Reimann and Bechara, 2010).

In order to simulate ambiguity decision scenarios in real life and further validate SMH through empirical research, Bechara et al. (1994) developed the Iowa gaming task (IGT). In this task, the size and probability of win and loss are systematically manipulated (Bechara et al., 1994, Bechara et al., 2000). Participants were asked to repeatedly pick up a card from the four decks representing different amounts for 100 trials, and then feed back the results of the selection (gain or loss). The ultimate goal was to obtain most gain or least loss. The two disadvantageous decks have a high immediate reward and also give higher levels of punishment (so leading to a net loss every 10 trials), whereas other two advantageous decks have low immediate reward and also give lower levels of punishment (so leading to a net gain every 10 trials). Participants only know that there are two advantageous decks and two disadvantageous decks, but they do not know which decks are advantageous or disadvantageous and the probability of loss or gain in these decks. Healthy participants must undergo a long process of exploration and step-by-step learning during the IGT (Bechara et al., 1996; Chiu et al., 2008). In this process, somatic markers generated by emotional components help to predict long-term positive or negative outcomes, especially before conceptualized explicit knowledge has been developed. Eventually, decision makers are shown a preference for advantageous decks than disadvantageous decks. However, some participants(e.g., patients with ventrolateral medial prefrontal cortex damage) always have difficulty making the right decisions, Even though they obtained conceptualized explicit knowledge in the later blocks of the IGT. Due to the lack of emotional-related somatic signals caused by damage to the medial prefrontal cortex, they were affected by short-term interests and still showed stubborn preference for advantageous cards, which ultimately led to poor performance in the IGT (Giustiniani et al., 2015).

Damasio first used the multichannel physiological recorder to measure the skin conductance response (SCRs) of participants in the IGT as a index of somatic markers, and thus explored and verified the physiological basis of the SMH. With the development of cognitive neuroscience technology, researchers began to explore the neural basis of the SMH with brain imaging techniques such as Functional Magnetic Resonance Imaging (fMRI) (Ernst et al., 2002). Neuroimaging studies of the SMH confirmed two key structures that triggers somatic markers. The first category is called the primary inducers, which is the somatic markers that triggers the amygdala produced by emotional events in the external environment; The second category is secondary inducers, which is the somatic markers that triggers the orbital frontal cortex (OFC) and the ventromedial prefrontal cortex (VMPFC) produced by information in memory, knowledge, and cognition (Bechara and Damasio, 2005). VMPFC and OFC are believed to be the core brain regions of the SMH, which can integrate emotional-related somatic signals from the periphery to the central nervous system to regulate and monitor cognitive processes of decision-making (Damasio, 1994, 1996). In addition, the neural structures of the SMH also include somato sensory and Island cortex, as well as the peripheral nervous system. The results of brain imaging studies provide support for further exploration of the physiological basis of the SMH. Subsequent researchers began using EEG and ECG as a complement to brain imaging and SCRs research. From the existing research, SCRs, EEG and ECG can quantify the emotional and feeling responses related to decision-making, which help to reveal the relationship between emotion-related somatic signals and decision-making, and is the most extensive physiological indicator for studying on SMH and ambiguity decision. However, there are still many controversies in the existing research results (see Table 1 and Wong et al., 2011; Chiu et al., 2018), and SMH has been questioned by many researchers. Therefore, the current study intends to review the existing research on the influence of the SMH on the IGT performance from three aspects of SCRs, EEG and HR, try to reveal the causes of inconsistent results, and explore propose possible future research directions. We hope to provide a basis for more accurately and completely revealing the neurophysiological mechanism of SMH.

**TABLE 1:** Main electrophysiological studies on decision-making under ambiguity in our review.

Authors	N	Participants	Task	Index	Electrophysiology Results
Bechara et al., 1996	19	12 healthy participants;	IGT	SCRs	•Healthy subjects generated an anticipatory SCRs prior to their selection of a deck. •Patient did not produce a similar anticipatory SCRs.
		7 patients with prefrontal damage	100		
Carter and Pasqualini, 2004	30	Healthy women	IGT	SCRs	•The amount of money gained and mean anticipatory SCRs were positively correlated. •No relationship was found between reactive SCRs and either reward or punishment.
			100		
Ottaviani and Vandone, 2015	445	Experts in economics and finance	IGT	SCRs	**•**The anticipatory SCRs during the first 40 trials were negative significant predictors of insurance purchase.
			100		
Mardaga and Hansenne, 2012	32	Healthy participants	IGT	SCRs	**•**More anticipative responses are recorded before disadvantageous than advantageous deck picking.
			100		
Fernie and Tunney, 2013	32	Post-graduate students	IGT	SCRs	•Most participants had knowledge sufficient to guide behavior after approximately 40 trials. •Their not find anticipatory physiological activity sufficient to differentiate between deck types in the period prior to acquiring this knowledge.
			100		
Gutbrod et al., 2006	19	8 healthy participants	IGT	SCRs	•Healthy participants acquired a preference for advantageous choices and generated large SCRs to high levels of punishment.
		11 patients with memory impairment	100		•The anticipatory SCRs to disadvantageous choices were larger than to advantageous choices. •This dissociation occurred much later than the behavioral preference for advantageous alternatives
Bianchin and Angrilli, 2011	16	Healthy men	IGT	ERP	**•**A greater negativity activated before picking from disadvantageous decks than advantageous desks in the right frontal sites. •N260 showed a significant Feedback effect, in which participants showed greater positivity for gains than for losses.
			150		
Giustiniani et al., 2015	20	Healthy subjects	IGT	ERP	•P200 was more positive for the favorable group than for the undecided group regarding the frontal electrodes, and P300 was more positive after a loss only in the favorable group. •Before choosing a disadvantageous deck, a more negative potential was present in the emotion-related right frontal sites.
			200		
Tamburin et al., 2014	48	24 normal subjects; 24 patients with cLBP	IGT	ERP	•The FRN amplitude in the Fz channel was higher to wins than losses in controls, while the opposite happened in patients.
			100		
Jang et al., 2012	13	Healthy students	IGT	ERP	•Greater FRN amplitudes were elicited in “loss” than “win” conditions. •Significant negative correlation between FRN amplitude and total number of selected advantageous deck was observed.
			300		
Di Rosa et al., 2017	36	21 were assigned to the younger group, 15 participants formed the older group	IGT	ERP	•The P300 amplitude was significantly reduced after negative feedback in older adults, compared with the younger ones. •The difference between the P300 amplitude elicited after positive and negative feedback was significantly larger in older than in younger adults, and this same difference is positively correlated with age.
			100		
Dong et al., 2016	34	17 high group participants (7 males, 10 females) and 17 low group participants WCST	IGT	ERP	•The P300 recorded from central and parietal regions when a bad deck appeared was larger in the high group participants than in the low group participants.
			600		•Losses evoked a larger FRN than wins in the high group, but the opposite result was found in the low group.
Schutter et al., 2004	18	Healthy participants	IGT	EEG	•Relative enhancement of resting alpha levels in the left prefrontal cortex (PFC) compared to the right hemisphere is associated with poor performance in IGT.
			100		
Giustiniani et al., 2019	20	Healthy participants	IGT	EEG	•The unilateralization of the resting alpha level of PFC is only related to risky behavior and has no direct relationship with performance in IGT.
			200		
Balconi et al., 2014	31	17 high reward sensitivity participants	IGT	EEG	•Compared with low reward sensitivity group, high reward sensitivity group have a less-sided alpha power, that is more left activity when selecting unfavorable cards.
		14 low reward sensitivity subjects	100		
Crone et al., 2004	96	Healthy students	IGT	SCRs	•Only performance group produced a larger SCRs before disadvantageous choices compared with advantageous choices. •SCRs rose following punishment in all samples.
			100	HR	•Only performance group produced a slower HR before disadvantageous choices compared with advantageous choices. •The main effect of feedback is significant which indicated that HR slowed down following punishment in all samples.
Lee et al., 2010	28	Healthy students	IGT	HR	•Subjects tended to make bad decisions rather than good decisions when the anticipatory HR slowed down.
			100		

*Each line refers to authors, index, the number of participants and major findings of the study.*

## Electrophysiological Indicators of SMH

### Skin Conductance Responses

Skin Conductance Responses (SCRs) are the most commonly used indicators for measuring emotion-related somatic markers in ambiguity decisions, which is to measure the electrical conductivity of the skin by applying a small constant voltage between the two points of the skin (Park, 2009). When the participants are stimulated to cause an emotional response, the electrical conductivity will change significantly (Boucsein, 2012). The higher the level of emotional initiation, the stronger the SCRs. In the IGT, researchers often focus on SCRs in two time periods: (1) The anticipatory SCRs before selection reflect the expected assessment of the outcome in subsequent decision. (2) The reactive SCRs in the feedback phase reflect the evaluation of the feedback outcome. Because SCRs have an latency period of 2–3 s. Therefore, the time window for reactive SCRs is generally set to 5 s after feedback, the time window of anticipatory SCRs is set to 5 s before selection, and the interval between two trials is set to 6 s (Bechara et al., 2000). Healthy participants showed higher sympathetic excitation and SCRs prior to select disadvantageous decks rather than advantageous decks (Bechara and Damasio, 2002; Bechara et al., 2002). This increase in anticipatory SCRs will unconsciously guide participants to avoid disadvantageous decks and select advantageous decks in subsequent decisions before forming conceptualized conscious knowledge (Bechara et al., 1996, 1997).

The early evidence for the SMH is mainly from clinical studies of the patients with VMPFC or amygdala damage but maintaining a normal intellect. These clinical studies have shown that the anticipatory SCRs will affect the patient’s performance in the IGT (Bechara et al., 1994, Bechara et al., 1996, 1999, 2000). Subsequent researchers have further expanded their research areas, mainly involving material addict, non-material addict, neurological and psychiatric patients, patients with hippocampus damage, and parkinson’s patients (Manes et al., 2002; Mapelli et al., 2014). The results of those studies are basically consistent with the conclusions of brain imaging studies. Individuals whose emotion-related brain regions are damaged are unable to produce anticiptory SCRs that characterize long-term negative outcomes in decision-making, which leads to impaired decision-making ability. Even if they gain conceptualized explicit knowledge, they are still stubborn in their preference for disadvantageous options (Giustiniani et al., 2015).

Simonovic et al. (2019) conducted a comprehensive mate analysis of the relationship between IGT performance and aSCR of healthy participants in existing studies, which provides an important basis and direction for future research on SMH and SCRs. Based on the effect sizes, author found that 11 studies reported overall aSCR correlates with successful performance on the IGT was small, significant differences, and 4 studies found medium and large effect sizes. The results show that the performance of healthy participants in IGT is closely related to aSCR. However, research on the relationship between aSCR and IGT success remains inconsistent over the past two decades. Mardaga and Hansenne (2012) found that the level of aSCRs of healthy participants positively predicted IGT performance. Similar research also found a significant positive correlation between IGT performance and overall aSCRs of healthy participants (Carter and Pasqualini, 2004; Wagar and Dixon, 2006; Guillaume et al., 2009; Werner et al., 2009; Miu et al., 2012), which indicates that overall aSCRs represent a good somatic signal. Larger good somatic signals will guide participants in subsequent decisions and lead to IGT success. However, the research by Ottaviani and Vandone (2015) gave the opposite result, they found that aSCRs reversely predicted IGT performance of healthy participants. That is to said, overall aSCRs represent a bad signal. Participants producing larger aSCRs will lead to worse IGT performance. This may be because two-thirds of the participants in this study were experts in economics and finance. Experts in economics and finance may perform differently than others in economic decision-making. In other studies, participants were generally not allowed to be professionals in economics and psychology.

In addition, whether there is a causal relationship between the success of IGT and aSCRs is also questioned. Some studies suggest that aSCRs represent a better IGT performance, but the success of IGT and aSCRs is caused by conscious knowledge (Maia and McClelland, 2004; Wagar and Dixon, 2006; Guillaume et al., 2009). For example, Fernie and Tunney (2013) found that participants had sufficient knowledge of task accidents to guide behavior after about 40 trials. Although the enhancement of aSCRs signal represents a better IGT performance, the relationship between aSCRs and IGT performance was not found until sufficient conscious knowledge was generated. Gutbrod et al. (2006) also found that preference for favorable cards appeared before the differences in aSCR occurred. These studies show that aSCR and IGT success are related, but may not be causal, and the enhancement of aSCRs signal may not be a necessary condition for IGT success. Conversely, some studies have found that healthy participants generate larger SCRs in the face of negative feedback during the feedback phase, which may guide the participants’ decisions in IGT. And when participants learn of such unexpected events, feedback on SCRs becomes less important (Suzuki et al., 2003; Wright et al., 2017). This indicates that healthy participants may have learned about IGT based on card feedback. Once conceptualized knowledge is formed, they can ignore somatic signals and directly guide subsequent decisions. These inconsistent findings may be due to their differences in IGT design. For example, compared to Fernie and Tunney (2013) and others’ studies, Bechara’s series of studies are more obscure in measuring conceptual knowledge. In addition, most of the samples in the existing studies are relatively small, which may lead to interference factors such as gender, age, personality, and cognitive abilities. Based on the existing research, we can speculate that there may be an interaction on the time axis between SCR, conscious knowledge about IGT, and IGT success. In the early days of IGT, feedback SCR was the main somatic signal that guided healthy participants to develop unconscious knowledge of each card (within 30 trials). Subsequently, the unconscious knowledge of the card will cause the expected somatic signal (aSCR) before the card selection, forming a “hunch” that guides decision-making at the unconscious level, and will also gradually form the conceptualization of IGT (10 to 80 trials). At this time, aSCR and conceptualized conscious knowledge may be a mutually reinforcing relationship. When conceptualized knowledge is formed (60 to 100 trials), healthy participants can use conceptualized knowledge to guide decision-making. At this time, aSCR signals and feedback SCR signals are no longer important, but may still exist. Future research should increase the number of subjects, control the individual differences of the subjects, optimize the experimental design as much as possible (e.g., increase the sensitivity of IGT knowledge measurement), and conduct a more detailed analysis of each block to validate the timeline relationship between SCR, conceptual knowledge, and IGT success.

It is doubtful whether the enlargement of an absolute somatic marker can guide decision-making. It is important to select whether there is a difference between aSCRs before favorable cards and unfavorable cards. Previous studies have found that as IGT trials progress, healthy participants gradually gain a preference for favorable choices, and their aSCR for unfavorable cards is significantly greater than that for favorable cards (Bechara et al., 1996, 1999; Bechara and Damasio, 2002; Crone et al., 2004; Gutbrod et al., 2006; Wagar and Dixon, 2006; Guillaume et al., 2009). This is consistent with the original hypothesis of SMH (Damasio, 1994). However, a review by Simonovic et al. (2019) found that the difference in aSCR between favorable and unfavorable cards was less significant. Furthermore, in many existing studies on SCR and SMH, researchers have reported overall aSCRs, but have not reported detailed differences in aSCRs between favorable and unfavorable cards (Simonovic et al., 2019). This suggests that some authors may have problems with publication bias. In addition, SCR is affected by the activation of neuropsychological, behavioral, and inhibitory systems that involve responses to punishment and frustration (Fowles, 1988). SCR is also related to the advantages of card selection and the successful execution of tasks (Denburg et al., 2006; Hinson et al., 2006). Therefore, future research requires more repetitive research on the difference between aSCRs for favorable and unfavorable cards.

Furthermore, disputes about SMH and SCRs have always existed (Chiu et al., 2018). include: (i) The reason for the increase in SCRs is not clear, it may be due to loss (Bechara and Damasio, 2002) or win (Tomb et al., 2002), or caused by the probability or magnitude of win or loss (Wilder et al., 1998; Shurman et al., 2005). For example, Chiu et al. in their series of studies found that healthy participants’ decisions in IGT are related to the profit and loss probability of cards, and healthy participants show a persistent preference for unfavorable cards B with low loss frequency. However, the series studies did not record details of aSCRs. (ii) There may be multiple mechanisms that regulate both IGT performance and anticipatory SCRs production. Anticipatory SCRs may only be a part of the decision outcome, not the cause. Existing studies cannot confirm the causal relationship between anticipatory SCRs and task performance. (iii) Due to the relatively slow time course of SCRs signals, it is difficult to separate reactive SCRs and anticipatory SCRs in the next trial, which leads to a drop in the accuracy of the results (Amiez et al., 2003). In sum, IGT performance is significantly correlated with expected SCRs, but future research still requires more ingenious experimental design and careful analysis to support the SMH, and electrophysiological measures with high temporal resolution (e.g., EEG, HR) will be complementary to SCRs.

### Event-Related Potential

Electroencephalography (EEG) has higher temporal resolution, which provides direct access to neuronal signals compared with SCRs, and measured by Event-related Potential (ERP) (Michel and Murray, 2012). Therefore, researchers have shifted their focus to using EEG to record somatic signals in ambiguity decision making in recent years. Similar to SCRs, EEG research also focuses on the physical responses in the two stages of before selection and after feedback. Common ERP indicators include Decision Preceding Negativity (DPN), P300, and P200, P300, and feedback-related negativity (FRN) after result feedback. DPN generally selects the negative wave in the 0-500ms time window before the decision (Bianchin and Angrilli, 2011), which reflects expectations of card outcomes (Bianchin and Angrilli, 2011; Cui et al., 2013; Giustiniani et al., 2015). P300 before decision-making is a forward wave that peaks at a time window of 350-500ms after the stimulus appears (Maurage et al., 2007; Kraus and Horowitz-Kraus, 2014), and is a sign of attention and working memory. P200 and P300 in the feedback phase are late positive waves appearing in the time window of 150∼250 ms and 250∼600 ms after the feedback result, whose average latency is about 200 ms and 300 ms, respectively. P200 is generally considered to be an early evaluation of the results, using a binary classification method with good or bad results (Gehring and Willoughby, 2002; Hajcak et al., 2006; Schuermann et al., 2012). P300 reflects performance monitoring and behavioral adaptation (Schuermann et al., 2011; Cui et al., 2013; Ferdinand and Kray, 2013). This process is related to both the potency and size of the feedback (Ferdinand and Kray, 2013). FRN is a negative wave that appears during 200 ∼ 350 ms after the feedback result is presented (Wang et al., 2016), which reflects the early feedback evaluation of a binary good and bad classification, the degree of violation of the subject’s expectations (Alexander and Brown, 2011; Schuermann et al., 2011), and the process of implicit learning through feedback (Cui et al., 2013; Balconi et al., 2015).

Electroencephalography study using ERP technology to explore SMH found that, compared to favorable cards, unfavorable cards would induce greater pre-decision P300 and DPN. Cui et al. (2013) found that healthy participants induced greater DPN amplitude before choosing unfavorable cards (compared to choosing favorable cards). Furthermore, healthy participants with a higher average amplitude of overall DPN performed better in IGT. In addition, Bianchin and Angrilli (2011) found that the DPN difference between unfavorable and favorable cards was unilateralized in the brain area, and only appeared in the right prefrontal cortex. This is consistent with the results of previous brain imaging studies. Activation of the right prefrontal cortex has important roles in emotional expression (especially negative emotions) and control (Coan and Allen, 2003; Wager et al., 2008). These studies show that the somatic signals generated by the activation of the right prefrontal cortex can help participants’ learning process and guide participants to avoid adverse cards in subsequent decisions. However, Giustiniani et al. (2015) suggested that DPN may not be a key factor influencing whether healthy participants can perform well in IGT, and DPN amplitude cannot predict participants’ performance in IGT in their study. The reason why the two come to different conclusions may be due to different experimental paradigms. The participants need to make a play / pass decision on one of the four decks preselected by the computer on each trial in the study by Cui et al. (2013), where participants’ DNP reflects the EEG response caused by a particular card. The study by Giustiniani et al. (2015) is a standard IGT computer version, which participants are free to choose cards among four non-different cards. Four simultaneous non-different cards will attract participants’ attention, which may lead the DPN amplitude change caused by the final selected card is covered up. ERP research in the feedback stage found that participants produced ERP components (FRN, P200 and P300) with different amplitudes when facing positive and negative feedback. The difference in the amplitude of these ERP components in the two feedback situations can guide participants to correctly distinguish favorable and unfavorable cards in subsequent trials, so as to have better performance in IGT. However, previous studies have failed to reach a consistent conclusion. Jang et al. (2012) found that compared to positive feedback, negative feedback produces a larger amplitude FRN, which showed that FRN reflects ACC activities related to the evaluation of positive and negative feedback results. Moreover, there is a significant correlation between FRN amplitude and IGT performance, which indicates that the evaluation process reflected by FRN can be used to adjust subsequent decision-making behaviors. Subsequent research also found that the FRN amplitude difference during positive and negative feedback remained stable over age (Di Rosa et al., 2017). However, Tamburin et al. (2014) found that positive feedback produced a slightly larger FRN amplitude of the healthy control group than negative feedback, but the difference was not significant. The opposite happened in chronic low back pain (cLBP) patients, and the FRN amplitude in negative feedback was significantly higher than that in positive feedback. The authors believe that clbp patients seem to invert the correct placement of feedback according to the good vs. bad outcome basic classification. The absence of FRN effect in the healthy control group may be due to the inclusion of some relatively older participants in the control group (West et al., 2014), or the personality profiles and / or genetic variables (Mueller et al., 2014), and may be affected by unmeasured reward sensitivity (Balconi et al., 2015). It may also be because there is a slight difference in the IGT of the two studies. In the Tamburin et al. (2014) task, participants were told that there were two good cards and two bad card, which lead healthy participants gaining a part of the knowledge about IGT cards in advance, and there was some psychological preparation in evaluating the results of positive and negative feedback, so there was no significant difference in EEG activity. But Jang et al. (2012)’s study did not report whether participants were informed of this information.

Tamburin et al. (2014) found that the P300 amplitude of healthy participants during positive feedback was significantly greater than the P300 amplitude of negative feedback. This indicates that P300 is a kind of positive feedback (that is, the amplitude of the positive feedback is greater than the negative feedback), which reflects the realization of the expected goal (Ferdinand and Kray, 2013). Therefore, the study showed that at an advanced stage of outcome processing, healthy participants were able to distinguish between positive and negative outcomes, using the experience of previous trials to guide subsequent decisions. However, Giustiniani et al. (2015) found that, compared to positive feedback, healthy participants who formed a successful strategy in IGT had stronger activation of the frontal medial gyrus when processing negative feedback, and produced a larger P300 amplitude. The discrepancy between the results of the two studies may be due to the difference in the research paradigms adopted by the two studies. In the study of Tamburin et al. (2014), the number and potency of the feedback results were presented simultaneously, while in Giustiniani et al. (2015)’s study, the card amount was presented before feedback on the potency. The P300 in the feedback may not only reflect the card’s potency, but may also be motivated by the card amount presented in advance. Moreover, healthy participants were further subdivided into forming a successful strategy group, an unsuccessful strategy group and an undecided group in the study by Giustiniani et al. (2015). Differences within the healthy participant group are frequent, and studies have reported a failure rate of 55% of IGT in healthy people (Mapelli et al., 2014). Therefore, future research should be further subdivided into groups based on the performance of healthy participants to conduct more detailed analysis.

In addition, existing studies have also found that somatic signals are also reflected on the EEG spectrum. For example, alpha activity is considered to be a measure of the inactivity of the cerebral cortex and can provide information about the state of brain activation in the opposite way (Harmon-Jones and Allen, 1997; Cooper et al., 2003), and IGT is sensitive to tonic / stable physiological and psychological correlates of personality. Therefore, alpha activity has also become a potential indicator for measuring SMH. Schutter et al. (2004) found that the relative enhancement of the resting alpha level of the prefrontal cortex (PFC) in the left hemisphere was associated with poor performance in IGT compared to the right hemisphere. Because alpha is a counter-indicator, the study suggests that the unilateral advantage of right hemisphere activation can lead to the failure of IGT. This is in contradiction with the traditional emotional electrophysiology model, that is to said, the processing of punishment and reward is considered to be right-side and left-side of PFC, respectively. This may be because the explanation of PFC unilateralization did not take into account the psychological activities involved, nor did it take into account the differences between actions in preparation and execution. Moreover, Giustiniani et al. (2019) research found that the unilateralization of the resting alpha level of PFC was only related to risky behavior, and not directly related to IGT performance. Therefore, whether the resting alpha level can be used as an indicator of somatic signals still needs further investigation. However, the discussion of the relationship between resting alpha level and IGT can reveal individual differences in the IGT, which helps explain some inconsistent conclusions in existing studies.

Previous studies have found that ERP components in the IGT are affected by many factors, such as cognitive ability, working memory, cognitive load, and reward sensitivity. Dong et al. (2016) found that, compared with the low cognitive ability group, the high cognitive ability group showed a larger amplitude of P300 within the time window of stimulus lock time, which may be caused by the low cognitive and abstract generalization ability and working memory ability of the low cognitive ability group, which can not form a physical signal to guide decision-making. In addition, the high cognitive ability group had a more negative DPN when selecting pass than play, while the low group showed stronger DPN amplitude for play, which indicates that the high cognitive group is more inclined to explore the rules of the card, so they tend to “play” to identify the rules as quickly as possible. In the feedback evaluation phase, compared with positive feedback, the high cognitive ability group caused a larger FRN during positive feedback, while the low cognitive ability group had the opposite result. This is because FRN is more sensitive to loss. The high cognitive ability group can conduct implicit learning through feedback results, while the low cognitive flexibility group has defects in feedback learning and concept formation, which leads to the lack of FRN effect. In addition, Balconi et al. (2014) used alpha band modulation to measure the IGT performance of individuals with different reward sensitivities. They found that compared with individuals with low reward sensitivity, individuals with high reward sensitivity produced a less-sided alpha power, that is more left activity when choosing a disadvantaged card.

Age also affects participants’ IGT performance and their brain activity. Di Rosa et al. (2017) found that compared with positive feedback, young people had greater P300 amplitudes when negative feedback, while older people had the opposite result. Older people seem less willing to shift their attention from positive to negative feedback during the feedback phase, which may be the reason for their poor performance on the IGT. As Frank and Kong (2008) argues that performance at probabilistic selection tasks like IGT reflects individual set-shifting ability, i.e., the capacity to shift the focus of attention among stimulus dimensions, while relying on reinforcement to guide decisions. However, the study did not find that age has an effect on the early components of feedback processing (FRN), which suggests that aging may affect only the later stages of feedback processing (P300), but has no effect on the early stages (FRN).

### Heart Rate

Heart rate (HR) is another important indicator for measuring SMH. The main advantage of HR is that HR is jointly regulated by the sympathetic nervous system and the parasympathetic nervous system compared to SCRs. Conversely, due to the sweat glands do not have parasympathetic involvement, SCRs only reflects the sympathetic nervous system response. Therefore, the measurement of HR can reflect a situation when parasympathetic nerve activity changes while sympathetic nerve activity remains unchanged (Mark et al., 1985; Hampton et al., 2007). The main measurement methods of HR are electrocardiogram (ECG) and pulse oximetry. The results measured by these two methods are highly correlated (Giardino et al., 2002; Selvaraj et al., 2008). Due to the high temporal resolution of the ECG, it can provide very accurate continuous HR information. Therefore, ECG is widely used in clinical practice and scientific research, especially in the analysis of heart rate variability (HRV). The pulse oximetry measures the pulsation waveform of the internal finger artery by calculating the penetration of red light and infrared light in the finger, thereby measuring the continuous HR. The pulse oximetry has the advantage of being safe and simple. However, the accuracy of pulse oximetry for measuring HR is relatively low compared to ECG (Wong et al., 2011).

Anticipatory HR slows down when individuals are prepared to deal with offensive or threatened events (Somsen et al., 1983). Similar conclusions have been found in research on the IGT. Crone et al. (2004) found that participants who performed better had a slower anticipatory HR before choosing a disadvantageous decks than choosing an advantageous decks in the IGT. Participants whose anticipatory HR has no significant difference between choosing a disadvantageous decks and advantageous decks performed poorly in the IGT. Lee et al. (2010) also found that participants’ anticipatory HR changes are related to subsequent different types of decisions. When anticipatory HR is slower, participants are more inclined to make disadvantageous decisions than to make advantageous decisions. It can be seen that anticipatory HR is a relatively stable somatic signal, which can predict and explain the performance in the IGT. When a disadvantageous choice causes a slower anticipatory HR, then an evasive signal is formed that directs the participant to avoid disadvantageous choices in subsequent decisions. In addition, Miu et al. (2008) have explored the effects of anticipatory HR on the IGT performance in individuals with different trait anxiety. They found that compared with low trait anxiety individuals, anticipatory HR of high trait anxiety individuals before choosing disadvantageous decks would decrease significantly in the IGT, which makes it difficult for high trait anxiety individuals to distinguish between disadvantageous and advantageous decks. This result is consistent with the poor performance of high trait anxiety individuals in the IGT. The study of the feedback results found that HR of outcome feedback was significant, whether performing well, medium or poor in the IGT. This indicates that the change in HR caused by the feedback result may only be an autonomous reaction to the different valence of the results, which cannot provide favorable guidance for subsequent decisions (Crone et al., 2004).

However, although the main measurement method of HR, ECG, has higher temporal resolution and sensitivity than SCRs. However, the usage rate of HR is still relatively low in the study of emotional influence decision-making. This may be due to the limitations of HR measurements. For instance, ECG is similar to SCRs measurement, requiring electrodes to be placed on the participant’s arm, which results in HR accuracy being affected by arm activity (Park, 2009). The pulse oximetry measures HR through pulsating waveforms, and there are more possible influencing factors (e.g., heat loss and vasoconstriction), so the accuracy is relatively poor. Future research should be combined with other electrophysiological techniques to compensate for the inadequacies of HR measurements to better reveal and explain the impact of emotions on decision making.

In addition to the three main somatic signals of SCRs, ERP and HR, which can explain the impact of emotions on ambiguity decisions. Other biological states (e.g., endocrine and immune responses) can also have an impact on decision making. Therefore, electrophysiological measurements of emotion and body state also include pupils, blood pressure, and neurotransmitters such as DA, 5-HT, NA, and Ach, which also have a large potential for application. Researchers have begun to do some exploratory research. For example, Bierman et al. (2004) use eye tracking to explain the process associated with the generation of somatic marker signals. The pupil dilation was found to be a physical signal that predicts IGT performance. When the participant looked at the last card on the unfavorable card, the pupil dilation predicted a poor overall IGT performance, while pupil dilation on gaze at the last favorable card predicts better overall IGT performance (Simonovic et al., 2017). Byrne et al. (2015) found that blink rate promoted the success of depression in patients with IGT. In addition, neurobiochemical studies have found that the activity of its neurotransmitter noradrenaline is also related to ambiguity signaling (Lavin et al., 2014), and high baseline levels of cortisol (a steroid hormone related to fear and behavioral inhibition) predict participants’ success on the IGT (Van Honk et al., 2003).

## Conclusion

### A Combination of Multiple Electrophysiological Measurement Techniques

After more than two decades of development, the use of electrophysiological techniques to measure emotion-related somatic signals has become an important method for exploring the neurophysiological mechanisms of ambiguity decision making. However, as mentioned above, many existing studies based on an electrophysiological technology have found some inconsistent conclusions due to their limitations. To date, few integrated studies have used multiple measurement techniques. In fact, the several main electrophysiological techniques have their own advantages and complementarity. If two or more of these measurement techniques are combined, it will help to complement and compare the indicators. For example, SCR is a long time course index in seconds, while ERP is generally a high time sensitivity index within 1 s. At the same time, measurement of SCR and ERP can obtain a complete somatic signals on the time course. as mentioned earlier, HR is regulated by the sympathetic nervous system and the parasympathetic nervous system, while SCRs reflect the response of the sympathetic nervous system (Mark et al., 1985; Hampton et al., 2007). Therefore, if SCRs and HR are measured at the same time, it will provide empirical support for the differential response and interaction of the sympathetic and parasympathetic nervous systems in the decision-making process, and also help to explain the impact of emotions on ambiguity decisions more comprehensively. Moreover, SMH is a neural framework that contains many different neural processes involved in the decision making process. Different types of electrophysiological indicators such as ERPs and SCRs are only one part of them, and the relationship between these indicators is not clear. For example, SCRs are controlled by the peripheral nervous system. ERPs mainly measure EEG activation in the cerebral cortex such as VMPFC, while VMPFC is thought to integrate the somatic signals of the peripheral and central nervous systems (Damasio, 1994, 1996). Should there be some connection between ERPs and SCRs? Previous studies have found that the level of autonomic excitation reflected by SCRs and HR is relevant in decision-making (Batson et al., 1999; Tomb et al., 2002; Campbell et al., 2004; Crone et al., 2004). However, few studies have clarified the specific relationship between these indicators. In future research, a variety of electrophysiological measurement techniques should be further integrated to explore the intrinsic relationship of various electrophysiological indicators, so as to comprehensively reveal the physiological mechanism of the SMH.

Moreover, combining electrophysiological techniques with brain imaging techniques would be a potential way to validate SMH. Electrophysiological indicators such as SCR and ERPs reflect the temporal progression of peripheral and central nervous system activation, while brain imaging techniques can provide more accurate spatial localization. Therefore, the integration of electrophysiological techniques and brain imaging techniques will help to explore the physiological basis and neural basis of the SMH at the same time, and understand the psychological significance of various indicators as a whole. The lack of evaluation of electrophysiological indicators in previous fMRI studies limits the interpretation of the data. For example, it is difficult to determine whether VMPFC activation reflects emotionally related autonomic responses or other cognitive processes. Therefore, integrating fMRI with electrophysiological measurements will help to gain a more comprehensive understanding of the neurophysiological mechanisms of the decision-making process. How to solve the compatibility problem between fMRI equipment and electrophysiological equipment is the core to carry out this integrated research. For example, electrophysiological signals (e.g., SCRs) are often affected by magnetic induction and radio frequency (RF) pulses in an fMRI environment (Abacherli et al., 2005), resulting in the appearance of artifacts, which is more frequently present in ECG measurements. Fortunately, researchers and equipment vendors have begun to use a built-in real-time filter, a low-pass filter and a linear convolution model to separate clean SCRs signals and achieved good results in recent years (Wong et al., 2011). Future researchers can use these techniques to conduct more integrated research on electrophysiological techniques combined with brain imaging techniques. It is worth noting that artifacts from the data obtained from these systems are common, so it is necessary to perform the corresponding pre-processing before performing statistical analysis.

## Author Contributions

FX conceived and designed the review. LH and FX wrote the manuscript. LH revised the manuscript.

## Conflict of Interest

The authors declare that the research was conducted in the absence of any commercial or financial relationships that could be construed as a potential conflict of interest.
